# Selection and validation of reference genes for RT-qPCR normalization of porcine alveolar macrophages (PAMs) for PRRSV studies

**DOI:** 10.1038/s41598-023-35873-3

**Published:** 2023-05-31

**Authors:** Dayoung Oh, Ward De Spiegelaere, Hans J. Nauwynck

**Affiliations:** 1 Laboratory of Virology, Department of Translational Physiology, Infectiology and Public Health, Faculty of Veterinary Medicine, Ghent University, Merelbeke, Belgium; 2 Laboratory of Veterinary Morphology, Department of Morphology, Imaging, Orthopedics, Rehabilitation and Nutrition, Faculty of Veterinary Medicine, Ghent University, Merelbeke, Belgium

**Keywords:** Cellular microbiology, Pathogens, Virology, Gene expression, Gene regulation in immune cells, Infection, Innate immune cells, Reverse transcription polymerase chain reaction

## Abstract

Porcine alveolar macrophages (PAMs) are widely used for in vitro studies of porcine respiratory viruses. Gene expression in these cells is altered by viral infection and cellular immune response. Real-time reverse transcription polymerase chain reaction (RT-qPCR) is a powerful technique for analyzing these changes. In order to obtain reliable quantitative RT-qPCR data and come to sound conclusions, stable reference genes are needed for normalization of target gene expression. In the present study, we evaluated the expression stability of nine reference genes in PAMs during cultivation and upon porcine reproductive and respiratory syndrome virus (PRRSV) inoculation. Using geNorm and NormFinder algorithms, we identified PSAP and GAPDH as the most stable reference genes under all experimental conditions. The selected reference genes were used for the normalization of CD163 expression under different conditions. This study demonstrates that selection of appropriate reference genes is essential for normalization and validation of RT-qPCR data across all experimental conditions. This study provides a new set of stable reference genes for future studies with porcine respiratory viruses in PAMs.

## Introduction

As a primary target cell, porcine alveolar macrophages (PAMs) are considered as a very relevant system for studying porcine respiratory viruses both in vivo and in vitro^1–6^. Gene expression assays for PAMs have been extensively used to investigate their role in the pathogenesis of porcine reproductive and respiratory syndrome virus (PRRSV) infections^7–11^.

The real-time quantitative reverse transcription polymerase chain reaction (RT-qPCR) technique is commonly used for studying gene expression profiles in numerous fields of scientific research due to its advantages in simplicity, sensitivity, and specificity^12^. Comparison of expression data from RT-qPCR requires appropriate normalization methods. The most common way for the expression normalization is using stably expressed reference genes. Generally, so-called housekeeping genes are considered the most reliable as reference genes^13^. However, the expression level of endogenous reference genes is variable in between different cell types, within the same tissue and in the same cell type under different condition^14,15^. To overcome this disadvantage, it is necessary to identify and validate optimal reference genes for each cell type and experimental conditions under investigation. Reference gene candidates need to be compared to verify the stability of their gene expression. Multiple algorithms have been developed to evaluate reference genes^16–20^. In pigs, reference genes have been evaluated in different cell lines^21^ and alveolar macrophages upon exposure to bacterial pathogenic molecules^15^. However, reference genes have never been validated in gene expression studies of PAMs during in vitro viral infections. In this study, reference genes specific for the PAMs have been selected in the context of a PRRSV infection by comparing nine selected genes from transcriptomic data and previously known housekeeping genes. The assessment of the stably expressed reference genes was performed by applying a combination of two algorithms, NormFinder and geNorm, which selected most and least stable genes were evaluated by target gene normalization.

## Results

### Amplification efficiencies of reference gene candidates

**Figure 1:**
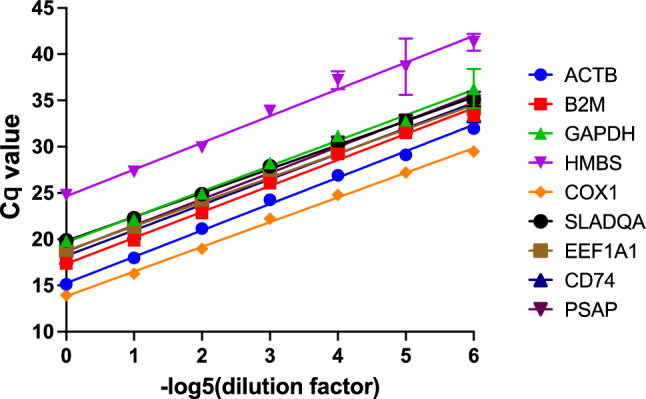
RT-qPCR primer efficiencies of 9 candidate reference genes.

The amplification efficiency calculated using the slope of the regression line ranged between 75 and 87% (Fig. 1, Table 1). The conventional linear regression method was compared with robust regression, which is less affected by outliers. The efficiency of PCR amplification was similar for both methods (Fig. S1). However, the efficiencies of reference gene candidates were consistent after running both regression methods on the results from the several independent replicates and using different qPCR machines. Moreover, standard curves generated by each gene of interest showed good linear relationships with correlation coefficients (R^2^) above 0.99 (Table 1) and specific amplification was confirmed by a single peak in the melting curve (Fig. S2). We also confirmed significantly high Cq values from RNA samples without a reverse transcription reaction (Table 1).

**Table 1:** PCR reaction efficiencies, correlation coefficient and Cq-value from cDNA and RNA templates.

Gene symbol	Efficiency (%)	Correlation coefficient (R^2^)	Cq value (cDNA) PAMs-16	Cq value (RNA) PAMs-16
*ACTB*	77	0.998	19.47 ± 0.61	33.60
*B2M*	79	0.996	21.35 ± 0.69	34.52
*GAPDH*	80	0.988	23.61 ± 0.61	31.68
*HMBS*	75	0.997	28.62 ± 0.37	32.12
*COX1*	83	0.994	17.66 ± 0.36	28.63
*SLA-DQA*	87	0.998	23.57 ± 0.56	33.95
*EEF1A1*	85	0.999	22.55 ± 0.45	33.26
*CD74*	83	0.999	22.32 ± 0.55	30.34
*PSAP*	78	0.994	22.79 ± 0.40	30.09

Cq-values from cDNA template are expressed as mean value of sixteen biological replicates ± standard deviation.

### Expression levels of candidate reference genes

**Figure 2:**
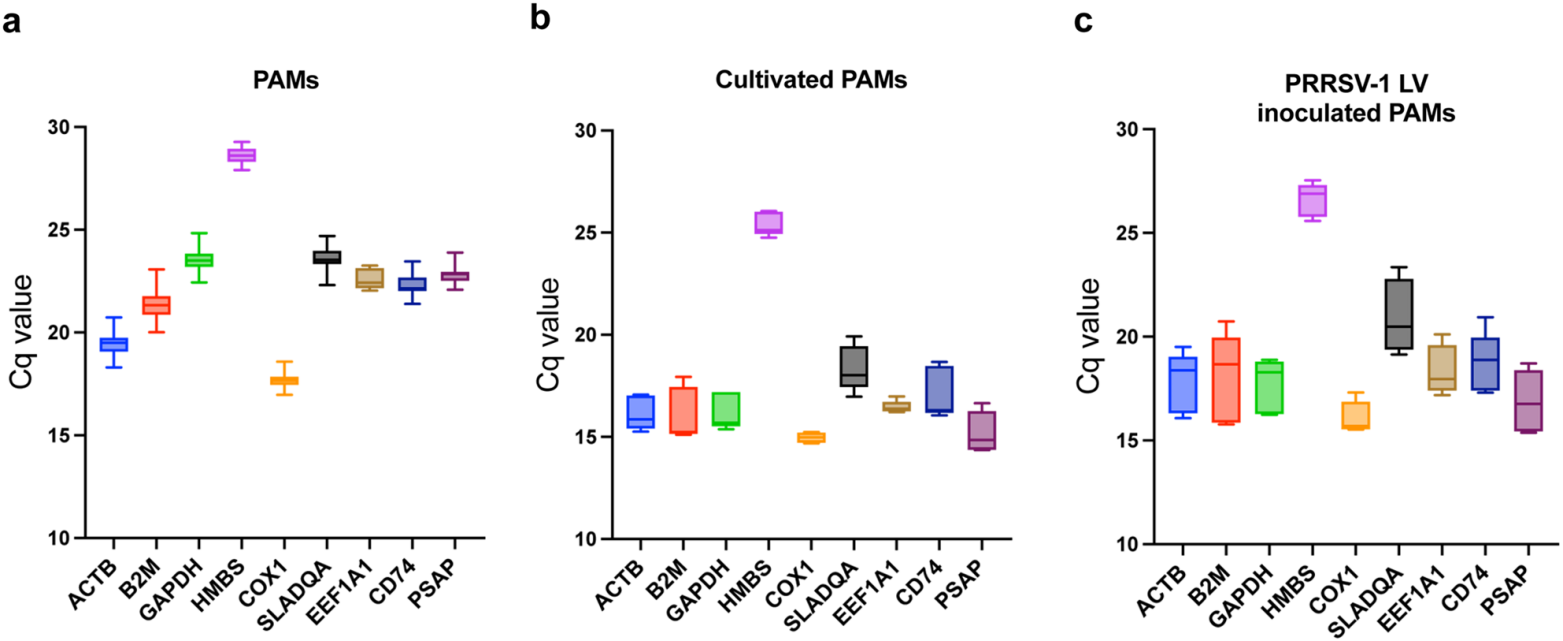
Range of Cq-values for candidate reference genes. The Cq values obtained from bronchoalveolar lavaged PAMs without culture (**a**), 24 h cultured PAMs (**b**) and PAMs 24 h post inoculated with PRRSV-1 LV (**c**). Box and Whisker plots show the Cq-value of the candidate reference genes. The grey boxes represent 25th to 75th percentile of the measurements. The black line in the box is the median and max and min Cq values are presented at the edge of whiskers.

We performed RT-qPCR reactions with the candidate reference genes in PAMs under different conditions to examine their expression profiles. Five randomly selected bronchoalveolar lavaged PAMs (PAMs-5) out of 16 biological replicates (PAMs-16) were used to study the expression of candidate reference genes after 24 h of cultivation (cultivated PAMs) or at 24 h after PRRSV infection (LV-inoculated PAMs). Candidate genes were expressed at varying levels under certain conditions. In all three groups, COX1 showed the highest expression among biological replicates (Cq value, PAMs-16: 17.66 ± 0.36, PAMs-5: 17.58 ± 0.29, cultivated PAMs: 14.97 ± 0.26, and LV-inoculated PAMs: 16.09 ± 0.77, respectively). (Fig. 2, Tables 1 and 2). The lowest expression was observed for the HMBS gene in all four groups (Cq value, PAMs-16: 28.62 ± 0.37, PAMs-5: 28.67 ± 0.33, cultivated PAMs: 25.40 ± 0.58, and LV-inoculated PAMs: 26.61 ± 0.81). The range of Cq value from the PAMs-16 and PAMs-5 groups was less variable compared to the cultivated and LV-infected PAMs groups (Fig. 2a and Table 1). The mRNA expression level of candidate genes was more fluctuant under different experimental treatments. The Cq value of nine candidate genes increased by an average of 1.67 (standard deviation: 0.43) in the LV-inoculated group, indicating that the expression of the corresponding genes decreased after PRRSV-1 LV inoculation (Fig. 2b, c).

**Table 2:** Cq-value in different conditions.

Gene symbol	Cq value PAMs-5	Cq value Cultivated PAMs	Cq value LV inoculated PAMs
*ACTB*	19.46 ± 0.23	16.13 ± 0.83	17.77 ± 1.45
*B2M*	21.36 ± 0.58	16.04 ± 1.29	17.96 ± 2.15
*GAPDH*	23.42 ± 0.27	16.20 ± 0.90	17.64 ± 1.31
*HMBS*	28.67 ± 0.33	25.40 ± 0.58	26.61 ± 0.81
*COX1*	17.58 ± 0.29	14.97 ± 0.26	16.09 ± 0.77
*SLA-DQA*	23.79 ± 0.56	18.33 ± 1.13	20.91 ± 1.78
*EEF1A1*	22.48 ± 0.42	16.46 ± 0.30	18.36 ± 1.19
*CD74*	22.40 ± 0.52	17.08 ± 1.25	18.68 ± 1.46
*PSAP*	22.80 ± 0.14	15.20 ± 1.01	16.83 ± 1.49

PAMs-5: PAMs without cultivation and PRRSV inoculation, cultivated PAMs: PAMs after 24 h cultivation, LV inoculated PAMs: PAMs 24 h post inoculation with PRRSV-1 LV. Cq-values from cDNA template are expressed as mean value of five biological replicates ± standard deviation.

### Selection of optimal reference genes

The expression stability of genes was evaluated using two statistical algorithms, geNorm^16^ and NormFinder^17^, to select appropriate reference genes for PAMs.

**Figure 3:**
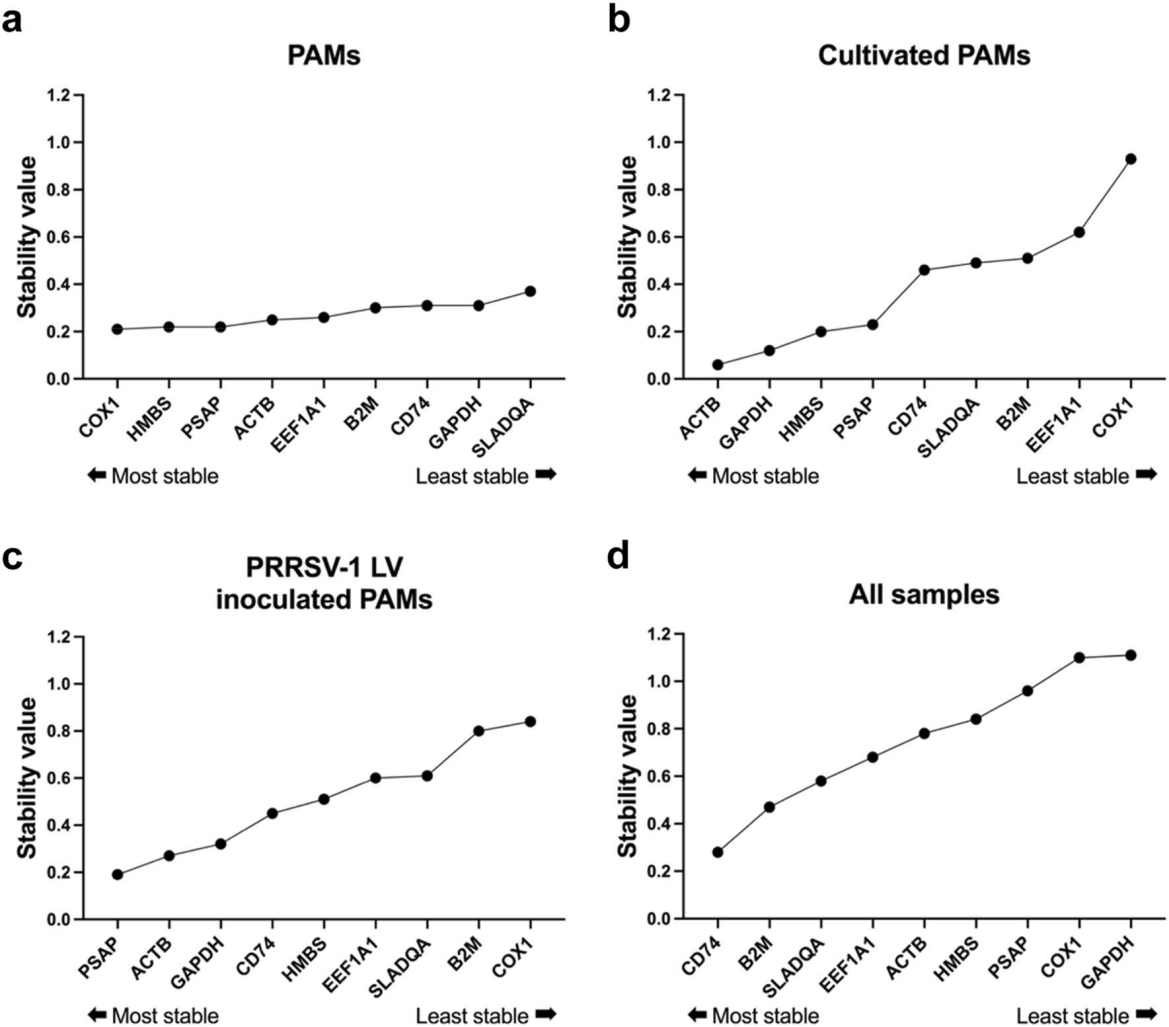
Ranking of candidate reference gene expression stability under different conditions based on a NormFinder analysis. PAMs isolated by bronchoalveolar lavage without cultivation and virus inoculation (**a**), PAMs cultivated for 24 h (**b**), PAMs inoculated with PRRSV-1 LV for 24 h (**c**), and all samples taken together (**d**). The calculated stability value is used to rank the reference genes in NormFinder. The lower the value, the higher the expression stability.

In NormFinder, stability values are assigned to each reference gene candidate by estimating the variation in expression, incorporating both variation within and between groups. Based on stability values, the top-ranked genes were COX1, HMBS, and PSAP as most stable genes in the PAMs-16 and PAMs-5 groups (Fig. 3a). At 24 h of cultivation, ACTB, GAPDH, and HMBS were the most stable genes (Fig. 3b). When PAMs were inoculated with PRRSV-1 LV strain, PSAP, ACTB, and GAPDH were the most stable genes (Fig. 3c). For all conditions, CD74, B2M, and SLA-DQA were the most stably expressed genes (Fig. 3d).

**Figure 4:**
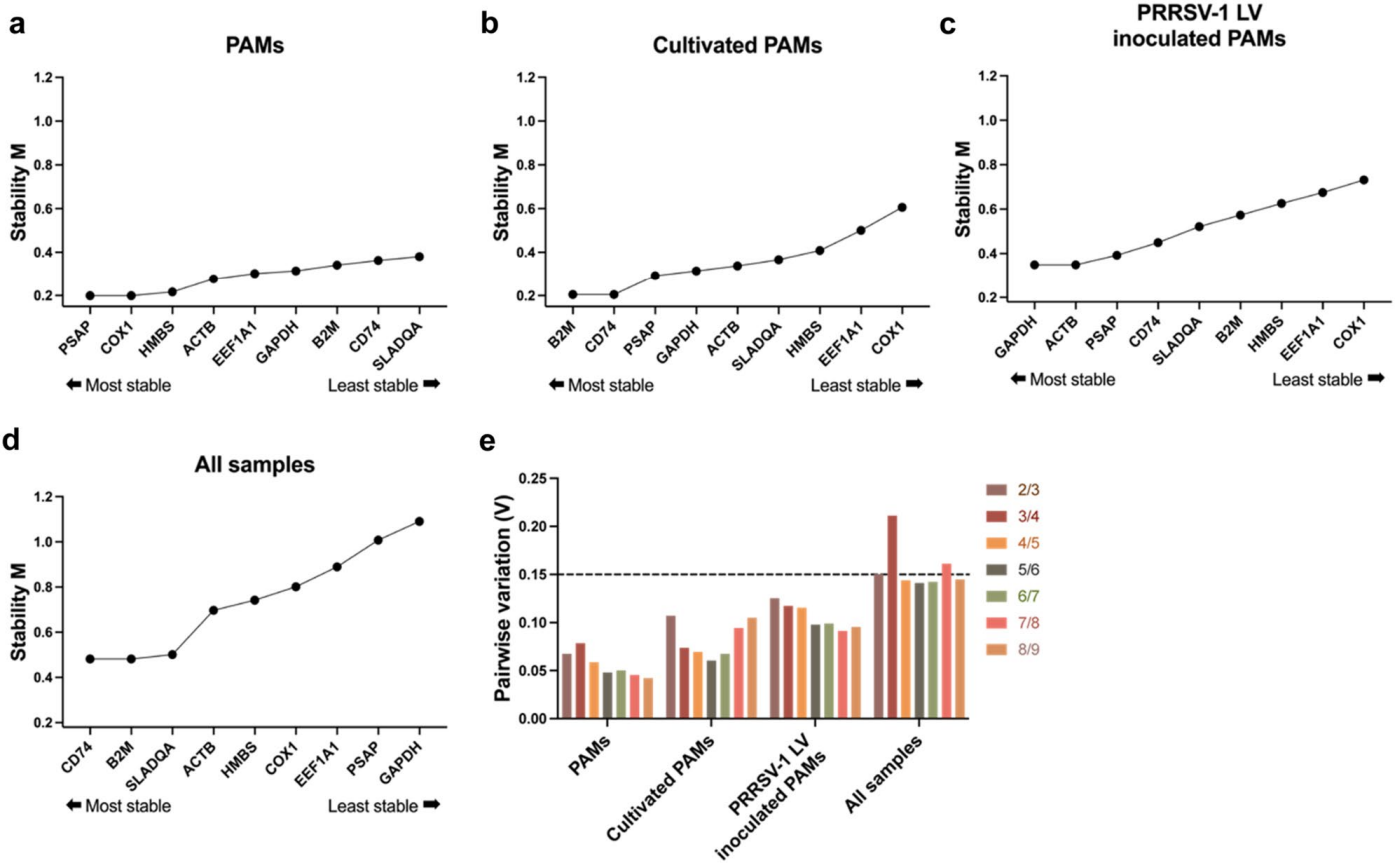
Ranking of candidate reference gene expression stability under different conditions based on a geNorm analysis. PAMs isolated by bronchoalveolar lavage without cultivation and virus inoculation (**a**), PAMs cultivated for 24 h (**b**), PAMs inoculated with PRRSV-1 LV for 24 h (**c**), and all samples taken together (**d**). Determination of the optimal number of candidate genes for normalization defined by pairwise variation (**e**). The stability parameter M is used to rank the reference genes in geNorm. The lower the value, the higher the expression stability.

GeNorm ranked the nine genes based on their gene expression stability measure “M”. A stepwise process of excluding the least stable gene allowed the genes to be ranked by M values. A lower M value indicates a more stable expression of a gene. The M value for all genes was below 1.5, which is considered as an acceptable expression stability. Variations in experimental conditions affected the most stably expressed genes. In the PAMs-16 and PAMs-5 groups, PSAP, COX1, and HMBS were the most stably expressed genes (Fig. 4a). At 24 h of cultivation, B2M, CD74, and PSAP were the most stably expressed genes (Fig. 4b). When PAMs were inoculated with PRRSV-1 LV strain for 24 h, GAPDH, ACTB, and PSAP were the most stable genes (Fig. 4c). For all conditions, CD74, B2M, and SLA-DQA were the most stably expressed genes (Fig. 4d). In NormFinder and geNorm anaylses, COX1 was identified as the most stable gene in BALF groups while this gene became the least stable gene during cultivation and viral inoculation. Both algorithms identified PSAP, ACTB, and GAPDH genes as the most stable genes in the PAMs upon cultivation and LV-inoculation.

GeNorm calculates the pairwise variation value (V) in comparison with two or more candidate gene combinations (Vn/Vn + 1). When the value of Vn/Vn + 1 is less than 0.15, n is considered as the optimal number of internal genes. Except the V3/4 and V7/8 in all conditions, the pairwise variations for all experimental conditions were below 0.15, indicating that two reference genes were sufficient to normalize the expression of the target gene (Fig. 4e).

### Normalization of target gene expression during PAM cultivation and PRRSV-1 LV infection

**Figure 5:**
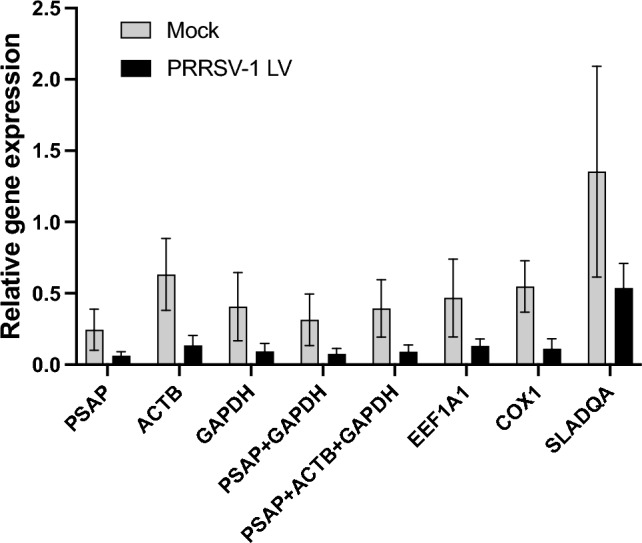
Relative expression levels of CD163 gene at 24 h cultivated PAMs and after 24 h inoculation with PRRSV-1 LV using six normalized reference genes. The relative gene expression levels of the target gene under different conditions were normalized to (i) the three most stable reference genes (PSAP, ACTB, and GAPDH), (ii) the combination of stable genes (PSAP + GAPDH or PSAP + ACTB + GAPDH) and (iii) the three least stable genes (EEF1A1, COX1, and SLADQA).

To evaluate the reliability of the selected reference genes, CD163 expression levels under different conditions were normalized using the three most stable reference genes (PSAP, ACTB, and GAPDH), a combination of stable genes (PSAP + GAPDH and PSAP + ACTB + GAPDH), and the least stable reference genes (EEF1A1, COX1, and SLADQA) (Fig. 5). In all cases, CD163 expression was higher in the 24 h cultivated PAMs than in the 24 h virus inoculated PAMs. The expression patterns of CD163 were similar in normalization with all selected reference genes except SLADQA. When the most stable reference gene, PSAP was used for normalization, the level of CD163 gene was the lowest and least variable between the biological replicates. However, the level of gene expression was the highest and most variable when the least stable reference gene, SLADQA, was used for normalization. Other two least stable genes, EEF1A1 and COX1, showed similar patterns of CD163 expression to the most stable reference genes. Normalization by gene pairs of PSAP + GAPDH and PSAP + ACTB + GAPDH showed a similar degree of CD163 expression and variation than when these genes were used alone.

## Discussion

Selection of proper reference genes is crucial to accurately obtain a reliable assessment of the target gene expression. As reference genes, endogenous genes, so-called housekeeping genes are typically used for normalization of RT-qPCR data. In fact, the expression of reference genes is greatly affected by cell type, tissue origin and experimental conditions. In pigs, alveolar macrophages are frequently used for studying the pathogenesis of viral infections and/or the immune response. It is a primary cell that easily adapts its gene expression which has been studied in both in vivo and in vitro studies^22^. Up till now, control genes were selected for studies on the role of cell and tissue type and the effect of bacterial pathogenic molecules, such as LPS and LTA in pigs^15,21^. However, reference genes in PAMs to follow the effects of a viral infection were not evaluated and validated yet. In this study, nine candidate reference genes were selected based on previous studies and own unpublished transcriptomic data on porcine lung macrophages. The stability of selected genes was analyzed under two experimental conditions: cell cultivation and PRRSV inoculation.

We first examined the amplification efficiency of candidate reference genes. The ideal amplification efficiency ranges from 90 to 110% suggesting that the number of molecules of the target sequence doubles in each replication cycle. Our primer efficiencies of candidate reference genes ranged between 75 and 87%. The amplification efficiency was not improved after performing various reaction conditions such as different primer concentrations and temperature gradient RT-qPCR (data not shown). In addition, despite excluding the effects of outliers of standard curves by a robust regression, no difference in efficiency could be demonstrated with the linear regression which considers outliers. However, single melting curves were identified in all candidate reference genes indicating that the specificity of the primers and the measured efficiencies were consistent over replicate runs. Next, we checked the Cq-value range for candidate reference genes. Our results clearly showed changes in expression level of reference genes between freshly isolated PAMs and cells under different conditions such as cell cultivation and viral infection. For all candidate reference genes, variation of Cq-values among the biological samples was the smallest in original PAMs (PAMs-16 and selection PAMs-5) without cultivation or viral infection. COX1 showed the highest expression and HMBS showed the lowest expression in all experimental conditions. However, expression levels of reference genes among the biological samples fluctuated in PAMs after cultivation or viral infection. In pigs, a number of research papers have been published in recent years examining endogenous control genes for normalization of gene expression data in different cell types, various tissues, and cell lines^15,21,23–28^. Gu et al.^29^ performed an extensive study for the validation of 20 endogenous control genes in 56 tissue types in pigs. Despite this, none of the studies provided a comprehensive selection of internal reference genes that remain stable in all tissue and cell type. Needless to say that it is very difficult to find stable reference genes under different experimental or infectious conditions. In our study, we therefore applied two algorithms to assess the stability of candidate reference genes. In both geNorm and NormFinder analyses, the stability ranking was completely changed before and after manipulations of the PAMs. GeNorm and NormFinder analysis showed that COX1 is the least stable gene in PAMs during cultivation and viral infection while this gene was the most stable gene in the non-cultured BALF group. It has been reported that infection of a highly pathogenic PRRSV strain on PAMs affects COX1 gene expression^30^. Unstable expression of COX1 gene upon PRRSV inoculation as shown in our study was in agreement with this study. In the original PAMs group (before cultivation/inoculation), although the order was different, PSAP, COX1, and HMBS genes appeared as the top three stable genes while SLADQA and CD74 appeared as unstable genes in both analyses. However, after 24 h of cultivation, the most stable genes were ACTB, GAPDH, and HMBS in NormFinder analysis while geNorm analysis ranked B2M, CD74, and PSAP as most stable genes. COX1 and EEF1A1 genes were ranked as the least stable genes in both analyses. Twenty-four hours after PRRSV-1 LV inoculation, both analyses identified PSAP, ACTB, and GAPDH in different orders as the most stable genes and COX1 as the least stable gene. In both NormFinder and GeNorm analysis of the combined gene expression data from all conditions, CD74, B2M, and SLADQA gene were found to be the most stable while GAPDH and PSAP genes were the least stable genes.

Taken together, PSAP, ACTB, GAPDH, EEF1A1, COX1, and SLADQA genes were used for the normalization of CD163 gene expression. CD163 mediates PRRSV infection in PAMs and its expression is regulated by the innate immune response to PRRSV infection^31^. Our results showed that the expression of CD163 was affected by both conditions (cultivation and infection). The variation in the level of CD163 was highest using SLADQA as a reference gene followed by ACTB gene. ACTB gene is one of the most commonly used reference genes in gene expression studies of different mammalian species and cell lines^14,24,32^. In our study, this gene was ranked in the top four stable genes in both algorithms and in all conditions. However, ACTB showed the second highest expression variability in our experimental conditions demonstrating the importance of reference gene selection in studies using PAMs. SLADQA gene is in the family of MHC class II molecules playing a central role in the initiation of the immune response and the expression of this gene is changed upon viral infection. Indeed, the regulation of MHC II genes expression after infection by PRRSV, African swine fever virus (ASFV), influenza A virus and porcine circovirus type 2 (PCV2) has been reported previously^33–36^ which is in agreement with our study.

Except the ranking with all samples, PSAP gene was found in the top 4 stable genes for all conditions. As a reference gene, this gene showed the lowest variation of target gene expression. PSAP gene is a highly conserved lysosomal protein involved in glycosphingolipid metabolism^37^. Sun et al.^38^ reported that specialized murine tissues, such as the Harderian glands and macrophages of the lymph nodes, lungs, splenic tissue, and thymus, showed extremely high levels of expression suggesting that the PSAP locus, a supposed “housekeeping” gene, is subject to distinct tissue- and cell-specific regulation of its expression. This study supports PSAP as the most stable gene for normalizing the target gene expression in PAMs under different experimental conditions in PRRSV research. In addition, our evaluation study showed the low variation of target gene expression normalized by the combination of PSAP and GAPDH genes. To ensure the confidence in RT-qPCR results, it is recommended to use more than two reference genes. Combination of PSAP and GAPDH genes exhibited intermediate stability, supporting their extended use as relatively safe reference genes.

In summary, we demonstrated changes in expression of candidate reference genes and expression stability under various experimental conditions accompanying PRRSV research using PAMs. Suitable normalization of PAMs during cultivation and PRRSV infection should be assured using geometric means of PSAP and GAPDH genes. We clearly showed how reference gene stability is impacted by the experimental condition, hence, a further revalidation of selected reference genes will be necessary for infection study using different PRRSV-strains and other viruses. This study provides a new set of reference genes for the gene expression study of PRRSV using PAMs.

## Methods

### Sample collection and cDNA synthesis

In this study, we used porcine alveolar macrophages (PAMs) that were isolated from 16 different animals between the years 2011 and 2022, and subsequently stored. The PAMs were isolated by bronchoalveolar lavage (BAL) from lungs of euthanized 3-week-old healthy conventional piglets from a PRRSV negative farm (Table S1). In brief, piglets were euthanized with 12.5 mg/kg body weight pentobarbital (Kela, Hoogstraten, Belgium) and lungs collected with trachea were lavaged with ice-cold Dulbecco’s phosphate-buffered saline (DPBS). BAL fluid from each animal was separately filtered through sterile gauze and cells were centrifuged at 4 °C for 10 min at 400×*g*. Cell pellets were washed twice with ice-cold DPBS by centrifugation at 4 °C for 10 min at 400×*g* and resuspended in complete RPMI-1640 media (Gibco, Paisley, UK) supplemented with 10% fetal calf serum (Sigma-Aldrich, St. Louis, MO, USA), 1 mM sodium pyruvate (Gibco, Paisley, UK), 1% non-essential amino acids (Gibco, Paisley, UK), 0.05 mg/mL gentamicin (Gibco, Paisley, UK), 0.1 mg/mL streptomycin (Gibco, Paisley, UK), 100 U/mL penicillin (Gibco, Paisley, UK). Upon collection, cells were stored in liquid nitrogen. Total RNA extraction was performed on 10 million PAMs with RNeasy mini kit (Qiagen, Hilden, Germany) according to the manufacturer’s instructions. RNase free DNase set (Qiagen, Hilden, Germany) was treated to remove DNA contamination. RNA concentration and purity were assessed with a NanoDrop 2000 spectrometer (ThermoFisher Scientific, Boston, MA, USA). The OD 260/280 ratio of the samples ranged between 2.02 to 2.08. The concentration of the samples ranged between 161.9 and 465.8 ng/μL. RNA integrity was verified by the automated electrophoresis system using Experion RNA StdSens analysis kit (Bio-rad, Temse, Belgium) according to the manufacturer’s protocol. Our samples showed good RNA integrity ranging from RNA quality index 9.4 to 10.0.

Approximately, 100 ng of RNA from each sample was converted to cDNA in the subsequent 20 μL RT reaction with the SuperScript IV first-strand synthesis system kit (Invitrogen, Vilnius, Lithuania) containing a random hexamer primer. cDNA was stored at −20 °C until further experiment. Sixteen RNA samples from the original PAMs were pooled. cDNA was synthesized from the pooled RNA samples, each 16 RNA samples originated from the original PAMs, and five RNA samples from each cultivated PAMs and LV-inoculated PAMs.

### PAMs culture and virus inoculation

Five samples were randomly chosen out of sixteen previously isolated PAMs and were used for virus inoculation. The PAMs were seeded at a concentration of 8 million cells/well in a 6-well plate. Cells were cultivated in 3 mL of complete RPMI-1640 medium in each well. PRRSV-1 subtype 1 Lelystad (LV) strain, that was passaged 14 times in PAMs, was used in this study. After 24 h pre-incubation at 37 °C with 5% CO_2_, cells were inoculated with either complete media (mock) or LV at a multiplicity of infection (MOI) of 0.2. Twenty-four hours post-inoculation (hpi), cells were washed three times with PBS and total RNA was extracted using RNeasy mini kit following the manufacturer’s protocol. The OD 260/280 ratio of the samples ranged between 1.92 to 2.09. The concentration of the samples ranged between 87.9 and 266.2 ng/μL. cDNA synthesis was performed as described above.

### Gene selection and primer design

Five reference genes with a high and stable expression in porcine macrophage subsets were selected based on transcriptome data from our laboratory (unpublished). Gene selection was restricted to the transcriptome data of macrophage subsets isolated by the fluorescence-activated cell sorting method. Primers were designed using Primer3 online software^39^. Candidate genes from transcriptome data were compared with four reference genes from the literature^23^. Specificity of the primer sequences was confirmed using a NCBI primer BLAST platform (Table 3).

**Table 3:** Information on the selected reference genes.

Gene symbol	Full name	Primer sequence (5’ → 3’)	Amplicon size (bp)	Ta (°C)	Accession number or reference
*ACTB*	β-actin	F: TCTGGCACCACACCTTCT R: TGATCTGGGTCATCTTCTCAC	114	60	Erkens et al.^23^
*B2M*	β-2-microglobulin	F: AAACGGAAAGCCAAATTACC R: ATCCACAGCGTTAGGAGTGA	178	60	Erkens et al.^23^
*GAPDH*	Glyceraldehyde-3-phosphate dehydrogenase	F: ACTCACTCTTCTACCTTTGATGCT R: TGTTGCTGTAGCCAAATTCA	100	57	Erkens et al.^23^
*HMBS*	Hydroxymethylbilane synthase	F: CTGTTTACCAAGGAGCTGGAAC R: TGAAGCCAGGAGGAAGCA	100	59	Erkens et al.^23^
*COX1*	Mitochondrially encoded cytochrome c oxidase I	F: CCGCAATGTCTCAATACCAAAC R: GTTGCGGTCTGTCAGTAGTATAG	122	57	NC_000845.1
*SLA-DQA*	MHC class II antigen	F: GATGTGCTCAACGACCTAGAA R: GTTCCAGAGAAGAGGTGAGAAG	92	57	NM_001114062.2
*EEF1A1*	Eukaryotic translation elongation factor 1 alpha 1	F: GGATGGAAAGTCACCCGTAAA R: GGACGAGTTGGTGGTAGAATG	86	57	NM_001097418.2
*CD74*	MHC class II invariant chain	F: ATCTGAAGCACCTCAAGAACA R: CAGCGAGTTCTTGCTCATTTC	101	57	NM_213774.1
*PSAP*	Prosaposin	F: GATCCTTGTGTACTTGGAGAGG R: AGGATGACAGGGAAGTAGGA	99	57	NM_001198919.1

*F* forward primer, *R* reverse primer, *bp* base pairs, *Ta* annealing temperature.

### Quantitative PCR

Quantitative PCR (qPCR) reactions were performed on StepOnePlus Real-Time PCR system (Applied Biosystems, Waltham, MA, USA) using PrecisionPLUS SYBR Green qPCR master mix (Primerdesign, Eastleigh, UK). To apply the same standard in the expression of reference gene candidates, standard curves were generated by five-fold serial dilutions of cDNA, which was synthesized by the pooled RNA samples. To analyze expression changes under the experimental condition, we conducted qPCR reactions using cDNA samples synthesized from 16 individual RNA samples as well as from five RNA samples each of the cultivated and the LV-inoculated PAMs. All qPCR reactions were carried out in the 96-well microtiter plate containing pooled cDNA standards for the standard curves, each cDNA sample and no-template control to check for contamination. Each reaction consisted of 3 μL cDNA, 300 nM forward and reverse primers, 5.8 μL DNase/RNase free-water and 10 μL 2× master mix. ROX was included in the master mix as a reference dye. Thermal cycling was initiated at 95 °C for 2 min followed by 45 cycles of 10 s at 95 °C and 1 min at the optimal annealing temperature for each pair of primers (Table 3). After amplification, a melting curve was generated to verify amplification specificity. The reaction for each sample was run in independent triplicates.

### Evaluation of primer efficiency

To evaluate amplification efficiencies, Cq-value from each serial dilution and each triplicate was analyzed by comparing linear and robust regression methods^40^ in Rstudio. The concept of robust regression by Trypsteen et al.^40^ considers a poor primer efficiency caused by outliers at the extreme of the dilution series. A robust regression estimator is less prone to outliers and is often more precise than a standard linear regression.

### Assessment of reference gene stability

To assess the stability of the selected reference genes, data were analyzed using the StepOne software v2.3 (Applied Biosystems, Carlsbad, CA, USA). The amplification efficiency, R^2^ values, melting curve and Cq-value for each gene of interest were exported to Microsoft Excel (version 16.72, Microsoft Redmont, WA, USA). The average Cq-value of each triplicate was analyzed using geNorm^16^ and NormFinder^17^ in Rstudio to evaluate the stability of the candidate reference genes. GeNorm M calculates the average pairwise variation between a particular gene and all other control genes in order to determine the stability of the reference genes. Stepwise exclusion of genes with the lowest expression stability values (highest M values) resulted in the average expression stability value (M). The M value for genes below the threshold of 1.5 was considered stable. In NormFinder, genes were ranked according to their stability values based on the variation in their expression within and across groups. Influence of amplification efficiency of each reference gene was included in NormFinder and geNorm analyses. Using these two algorithms, the most stably expressed reference genes were determined.

### Evaluation of the selected reference genes

The relative expression levels of the CD163 gene was analyzed using the three most stable reference genes and the three least stable reference genes using Hellemans’ method^41^ considering the PCR efficiency and the geometric mean of the multiple reference genes. The primer specification of CD163 gene is listed in the supplementary Table 2.

### Statistical analysis

The average Cq value was calculated from 16 biological replicates of PAMs group and five biological replicates for the mock and PRRSV-1 LV inoculated groups and three technical replicates. Plotting data from RT-qPCR, geNorm, NormFinder, and relative gene expression were conducted in GraphPad Prism 9 (GraphPad, San Diego, CA, USA).

## Supplementary Information

Supplementary Information.

## Data Availability

The data that support the findings of this study are available on request from the corresponding author.
