# Supplementary material for: Selection and validation of reference genes for RT-qPCR normalization of porcine alveolar macrophages (PAMs) for PRRSV studies
Source: Sci Rep. 2023 May 31;13:8840. doi: 10.1038/s41598-023-35873-3 (PMC10232543; doi:10.1038/s41598-023-35873-3)
Supplement: Supplementary file 1 — Supplementary Information. [file 41598_2023_35873_MOESM1_ESM.pdf]

Selection and validation of reference genes for RT-qPCR normalization of porcine alveolar macrophages (PAMs) for PRRSV studies

Dayoung Oh[1], Ward De Spiegelaere[2*], Hans J. Nauwynck[1*]

[1]Laboratory of Virology, Department of Translational Physiology, Infectiology and Public Health, Faculty of Veterinary Medicine, Ghent University, Merelbeke, Belgium

[2]Department of Morphology, Faculty of Veterinary Medicine, Ghent university, Merelbeke, Belgium

*Corresponding author: correspondence should be addressed to H.J.N. (email: Hans.nauwynck@ugent.be) and W.D.S. (ward.despiegelaere@ugent.be)

Supplementary Figures

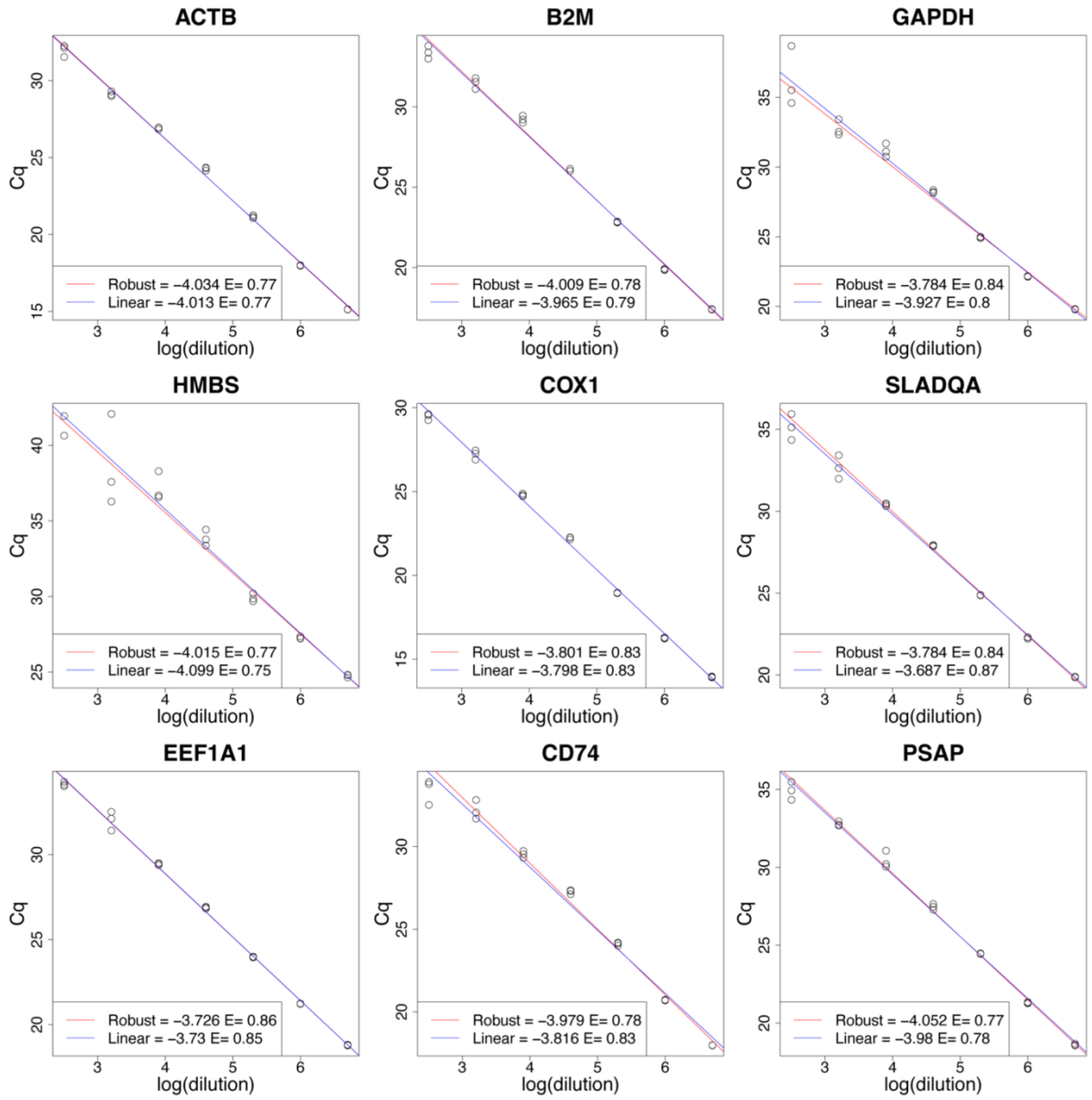

Supplementary Figure 1. Evaluation of amplification efficiency by comparing linear and robust regression methods. Robust: slope calculated by the robust regression method. Linear: slope calculated by the linear regression method. E: efficiency.

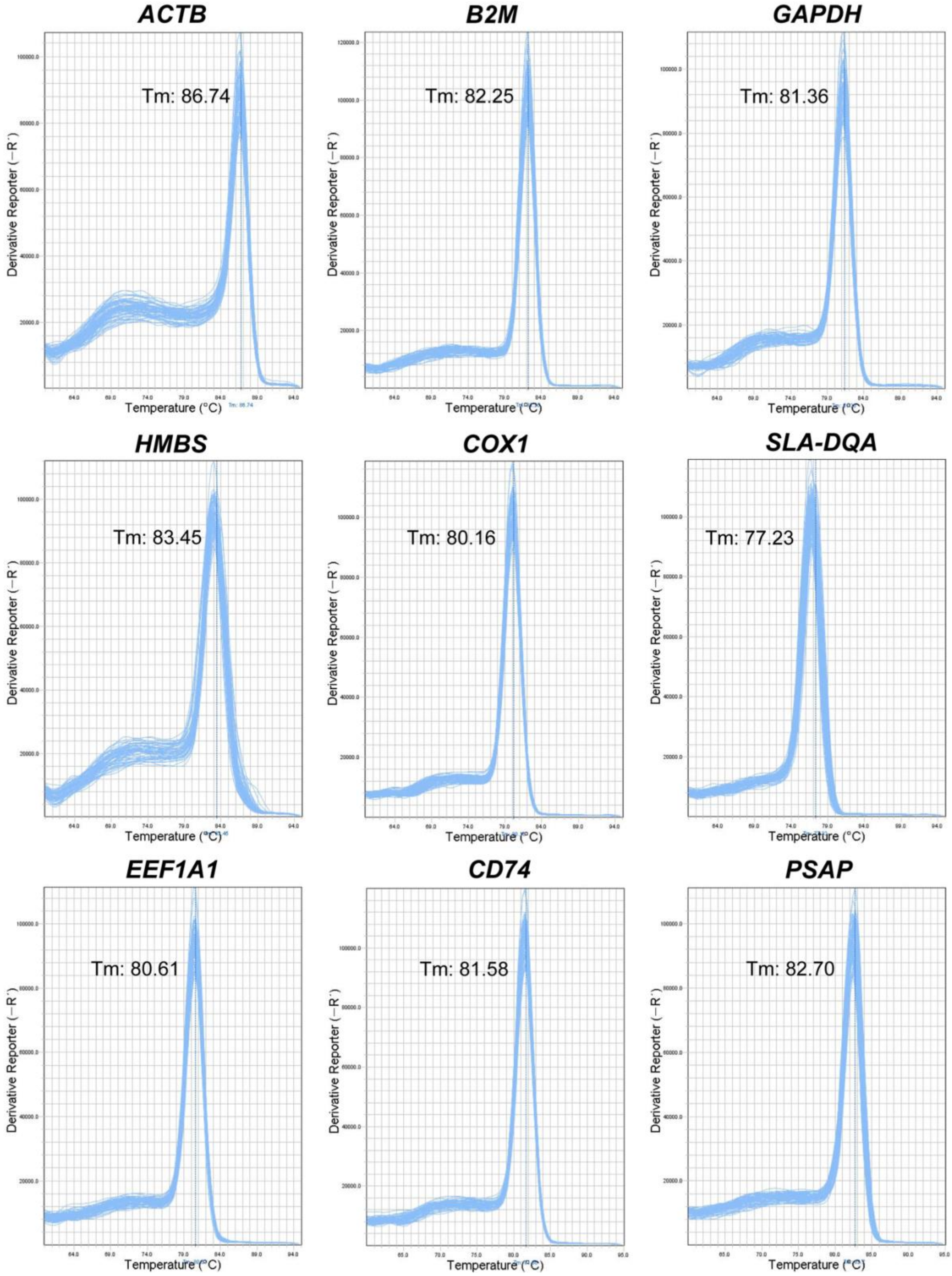

Supplementary Figure 2. Melting curve analysis for primer specificity. Single peaks were observed for each primer pair, which indicates that RT-qPCR amplification was specific and the primer dimers were absent. Tm: primer melting temperature.

Supplementary tables

Supplementary Table 1. Information of the PAMs used in this study.

PAMs number	Age of pig (week)	Cell isolation method	Year of isolation
1	3	Bronchoalveolar lavage	2022
2, 3	3	Bronchoalveolar lavage	2021
4*	3	Bronchoalveolar lavage	2019
5, 6	3	Bronchoalveolar lavage	2017
7*, 8, 9, 10*, 11, 12	3	Bronchoalveolar lavage	2014
13*	3	Bronchoalveolar lavage	2013
14, 15, 16*	3	Bronchoalveolar lavage	2011

* These PAMs were used in the PRRSV-1 LV inoculation experiment

Supplementary Table 2. Primer detail of target gene, CD163. F: Forward, R: reverse, bp: base pairs, Ta: annealing temperature, R^2: correlation coefficient of the corresponding standard curve.

Gene symbol	Primer sequence (5'→ 3')	Amplicon size (bp)	Ta (°C)	Efficiency (%)	R^2	Accession number
CD163	F: GTGACGTGCTCAGAAATTGC<br>R: AGACAGGCCAACAGAATGAC	104	57	103	0.999	NM_213976.1
